# Whole-Exome Sequencing for the Identification of Susceptibility Genes of Kashin–Beck Disease

**DOI:** 10.1371/journal.pone.0092298

**Published:** 2014-04-28

**Authors:** Zhenxing Yang, Yu Xu, Hongrong Luo, Xiaohong Ma, Qiang Wang, Yingcheng Wang, Wei Deng, Tao Jiang, Guangqing Sun, Tingting He, Jingchu Hu, Yingrui Li, Jun Wang, Tao Li, Xun Hu

**Affiliations:** 1 Psychiatric Laboratory & Department of Psychiatry, West China Hospital, Sichuan University, Chengdu, Sichuan, P R China; 2 State Key Laboratory of Biotherapy, West China Hospital, Sichuan University, Chengdu, Sichuan, P R China; 3 Biobank Center, West China Hospital, Sichuan University, Chengdu, Sichuan, P R China; 4 BGI-Shenzhen, Shenzhen, Guangdong, P R China; Wake Forest School of Medicine, United States of America

## Abstract

**Objective:**

To identify and investigate the susceptibility genes of Kashin–Beck disease (KBD) in Chinese population.

**Methods:**

Whole-exome capturing and sequencing technology was used for the detection of genetic variations in 19 individuals from six families with high incidence of KBD. A total of 44 polymorphisms from 41 genes were genotyped from a total of 144 cases and 144 controls by using MassARRAY under the standard protocol from Sequenom. Association was applied on the data by using PLINK1.07.

**Results:**

In the sequencing stage, each sample showed approximately 70-fold coverage, thus covering more than 99% of the target regions. Among the single nucleotide polymorphisms (SNPs) used in the transmission disequilibrium test, 108 had a p-value of <0.01, whereas 1056 had a p-value of <0.05. Kyoto Encyclopedia of Genes and Genomes(KEGG) pathway analysis indicates that these SNPs focus on three major pathways: regulation of actin cytoskeleton, focal adhesion, and metabolic pathways. In the validation stage, single locus effects revealed that two of these polymorphisms (rs7745040 and rs9275295) in the human leukocyte antigen (HLA)-DRB1 gene and one polymorphism (rs9473132) in CD2-associated protein (CD2AP) gene have a significant statistical association with KBD.

**Conclusions:**

HLA-DRB1 and CD2AP gene were identified to be among the susceptibility genes of KBD, thus supporting the role of the autoimmune response in KBD and the possibility of shared etiology between osteoarthritis, rheumatoid arthritis, and KBD.

## Introduction

Kashin–Beck disease (KBD) is a chronic, endemic osteochondropathy distributed mainly in a limited area from southeastern Siberia extending to northeast and southwest China[1]. According to the 2010 Health Statistical Yearbook of China, approximately 690,000 patients have been diagnosed with KBD, and more than 10 million people have a high risk of being affected in 366 counties within 14 provinces or autonomous regions of China[2]. Biogeochemical risk factors such as endemic selenium deficiency, high humic acid levels in drinking water, and contamination of mycotoxin-producing fungi in cereal have long been found to cause KBD [3]–[7]. However, the pathogenesis of KBD remains unknown. An increasing body of evidence suggests that environmental factors cannot solely account for the etiology of KBD [2].

Over the past few years, a number of studies provided evidence that KBD is a disease that is made complex by interactions between environmental factors and susceptible genes of the disease [3], [9]–[11]. For example, four candidate selenoprotein genes including GPX1, TrxR2, SEPP1, and DIO2 were analyzed for 161 KBD patients and 312 controls. Results indicated that the GPX1Leu allele can be associated with higher KBD risk [3]. Other studies on candidate genes have shown that genetic variants of human leukocyte antigen (HLA)-DRB1, which mainly causes osteoarthritis (OA) and rheumatoid arthritis (RA), were also associated with KBD [12]–[16]. Genome-wide gene expression analysis also suggested that chronic hypoxia-induced mitochondrial damage and apoptosis might be an important factor in the pathogenesis of KBD [4].

Although these previous studies may partially reveal several causes of KBD, no consistent conclusions have been drawn to identify the real pathogenic factors. The genetic contributions of this disease should be uncovered. Recent advanced analysis in whole-exome sequencing enabled an unbiased investigation of the complete protein-coding and flanking regions of the genome [5]. This technology has been successfully applied in the identification of Mendalian disorder [6] and has been proposed as a method for phenotypically complicated, genetically heterogeneous disorders when traditional linkage studies were unsuccessful [7].

In this study, 19 individuals from six multiple-affected KBD families were selected initially for whole-exome sequencing for the detection of functional genetic variation, which may be susceptible to KBD. The preliminary findings were then followed by association analysis in a larger independent case-control sample established to validate variants from significant sequencing signals and candidate genes derived from Kyoto Encyclopedia of Genes and Genomes (KEGG) pathway analysis.

## Methods

### Study Design and Subjects

The study was approved by the Ethics Committee of the West China Hospital of Sichuan University. All next of kin, care takers or guardians consented on the behalf of participants to provid written informed consent for their participation. We used the following criteria to evaluate whether the participants had the capacity to consent: Firstly, patients have the ability to understand; Secondly, patients have the ability to know reason; Thirdly, patients have the ability to make rational decisions. If participants failed to fill out the consent form more than twice, their guardians were asked to fill out the consent on the behalf of patients.

This study was designed with two stages, including whole-exome sequencing on family-based analysis and validation in an independent sample set. Six families with multiple KBD patients were recruited from the endemic Nai Dang Village, Jin Chuan County, Aba Autonomous Prefecture of Sichuan Province, China. Detailed information on these family samples is listed in Table 1. Patients were examined when they were > = 5-year old and were diagnosed with KBD based on the following symptoms: persistent pain; limitation of mobility; deformity of the fingers, toes, knees, ankles, wrists, interphalangeal joint, hips, or shoulders; specific changes on X-ray photography [8]; lack of other arthritis diseases such as RA, OA, or local inflammation; and lack of a history of trauma [1], [9]. All participants are Han Chinese.

**Table 1:** Detailed information on all sequencing samples.

Family No.	Individual No.	Sex (Male/Female)	Age	Affected (clinical grades of KBD)/Unaffected
Family 1	A0001 (Father)	M	69	Affected (I)
	A0002 (Mother)	F	68	Unaffected
	A0123 (Daughter)	F	44	Affected (II)
Family 2	A0169 (Father)	M	43	Unaffected
	A0170 (Mother)	F	39	Affected (I)
	A0171 (Son)	M	17	Affected (I)
Family 3	A0306 (Father)	M	54	Affected (I)
	A0307 (Mother)	F	51	Unaffected
	A0308 (Daughter)	F	22	Affected (I)
Family 4	A0463 (Father)	M	66	Unaffected
	A0464 (Mother)	F	63	Unaffected
	A0467 (Son)	M	35	Affected (I)
Family 5	A0521 (Father)	M	69	Affected (I)
	A0522 (Mother)	F	67	Unaffected
	A0523 (Son)	M	39	Affected (I)
Family 6	A0527 (Father)	M	49	Unaffected
	A0528 (Mother)	F	46	Unaffected
	A0530 (Son)	M	19	Affected (II)
	A0531 (Daughter)	F	17	Affected (I)

Note: Diagnosis and grading standards according to Moreno-Reyes, R et al[28]. I denotes mild, IIdenotes moderate, and III denotes severe.

In the validation stage, a total of 144 cases of KBD and 144 matched healthy controls were recruited from the same populations in other endemic villages in Jin Chuan County.

### Whole-exome Capturing and Sequencing

Peripheral blood (5 ml) was collected from all participants, and genomic DNA was extracted according to a standard phenol–chloroform procedure. NimbleGenSeqCap EZ Exome v2.0 was used for exome capture. The exome capture kits were purchased from the manufacturer [10]. Exome capture libraries were constructed following the manufacturers’ standard protocol with optimized parameters. Sequencing was conducted on Illumina HiSeq2000 as paired-end 90 bp reads (PE90) after indexing and pooling libraries. Each library was sequenced to a depth providing approximately 60-fold mapped coverage on target regions. Raw image files were processed by using the Illumina pipeline (version1.3.4) for base-calling and for the generation of a raw read set.

### Exome Capture Data Analysis

Adapter contamination and reads with low quality were identified and then removed before mapping. Reads were aligned to human genome reference GRCh37 by using SOAP (v2.21)[11]. Consensus genotypes were called for loci on target regions and nearby (200 bp upstream/downstream) by using SOAPsnp [12]. SNPs were extracted, and those with low confidence were removed. All the dataset were deposited in NCBI SRA, and accession ID is SRP035444.

### Validation Genotyping in a Larger Sample Set

44 SNPs from 41 genes were selected for validation. Among them, 12 SNPs enrichment in chromosome 19 and other regions show lower P value were selected firstly. There pathways shows more interesting associated with KBD after KEGG pathway analysis [13], and 12 SNPs in Focal adhesion, 4 SNPs in metabolic pathway, also 3 SNPs in regulation of actin cytoskeleton were validated further. In addition, previous studies on Kashin–Beck susceptibility loci [12], [17], [26]–[28] indicated that gene in immune system may associated with KBD. So, 3 SNPs in HLA-DRB1 and 10 SNPs present significant in current study of immune related genes validated further. In total, 44 SNPs from 41 genes (Table S1) were employed for the association test, with 288 samples from the same population.

The samples were genotyped by using MassARRAY [14] under the standard protocol from Sequenom. Both SNPs and samples with a call rate of <90% were excluded in the data analysis. Case-control association analysis was conducted based on the genotyping data by using PLINK1.07 (http://pngu.mgh.harvard.edu/purcell/plink). Bonferroni correction was used to reduce the false positive introduced by multi-test [15]. SNP on gene function annotation used SIFT tools (http://www.snp-nexus.org/).

## Results

### Exome Capture Data Analysis

A total of 124 gigabases (Gb) of DNA sequence were generated, with an average of 6.5 Gb per sample (Table 2). Each sample has approximately 70-fold coverage of the target regions, thus covering more than 99% of the target regions. For each sample, approximately 95% of the target regions with no less than 10-fold coverage were obtained. Results suggest that these regions are expected to have high-confidence genotypes.

**Table 2:** Exome capture statistics.

	Male	Female	Total
Sample size	10	9	19
Target region size per sample (bp)	44,234,141	44,131,461	
	**Average**	**SD**	**Total**
Raw data yield (Gb)	6.530	0.383	124.069
Data mapped to target region (Gb)	3.110	0.221	59.097
Mean depth of target region	70.40	5.05	1,337.54
Coverage of target region (%)	99.32	0.06	
Fraction of target covered > = 10X	95.12	0.51	
Fraction of target covered > = 20X	88.18	1.34	
Fraction of target covered > = 30X	78.78	2.35	
Fraction of target covered > = 50X	57.20	3.86	
Fraction of target covered > = 70X	41.44	1.63	
Rate of nucleotide mismatch (%)	0.22	0.02	

Among the 78,458,750 high-confidence genotypes, 90,197 high-confidence SNPs were obtained for each sample. All these SNPs were located at the 188,342 SNP loci. Among these SNP loci, 181,870 only had two alleles on autosomes. These SNPs were then used in the transmission disequilibrium test (TDT), in which 108 and 1056 SNPs have p-values of <0.01 and <0.05, respectively (Figure S1 and Table S4). The most significant p-value site distribution concentrated on the 19q13.2 (chr19: 38742846-54254388), which contains the selenoprotein V (SELV) and transfer RNA selenocysteine 1 (TRNAU1) gene. In this study, IL4R, CD22, CD82, CD163L1, CD180, IL12A, HLA-F-AS1, CD2AP, and HLA-DPB1 genes were found to show a positive association with KBD. KEGG pathway analysis (http://bioinfo.vanderbilt.edu/webgestalt/option.php) indicates that a large number of genes with significant statistical difference focused on three major pathways: regulation of actin cytoskeleton, focal adhesion, and metabolic pathways (Table S2).

### Validation in a Larger Sample Set

Demographic characteristics of the sample set showed no significant difference in gender distribution between patients with KBD (58 males and 86 females) and healthy controls (66 males and 78 females) (χ^2^ = 0.906, p = 0.34). No significant difference in age distribution was found between patients with KBD (59.98±11.13 years) and healthy controls (57.53±10.23 years) (t = 1.94, p = 0.053).

Out of 44 loci and 288 samples, 43 (97.7%) and 283 (98.3%) passed the aforementioned filter criteria of 90%[14]. Genotype distribution in both cases with KBD and controls did not deviate from the Hardy–Weinberg equilibrium. Analysis for single locus effects reveals that two SNPs (rs7745040 and rs9275295) in the HLA-DRB1 gene and one SNP (rs9473132) in the CD2AP gene had a significant statistical association with KBD. This result was obtained when SNPs were analyzed by genotype and allele frequencies (p-value <0.001 for all SNPs by analysis of either genotypes or alleles, Bonferroni adjusted significance level) (Table 3).

**Table 3:** Identification statistics.

ID	Gene	Chr	Position	Allele	F_A	F_U	P	OR
rs7745040	HLA-DRB1	6	32,664,332	T/C	0.08462	0.2405	1.42E-06	3.42
rs9275295	HLA-DRB1	6	32,663,391	A/G	0.08582	0.1866	6.74 E-04	2.48
rs9473132	CD2AP	6	47,522,357	A/G	0.2276	0.3619	6.49E-04	1.92

## Discussion

In this study, a number of possible genes were initially found to be associated with KBD, including genes of inflammatory factors, SELV and TRNAU1 genes, as well as genes derived from three related pathways (regulation of actin cytoskeleton, focal adhesion, and metabolic pathways) in whole-exome sequencing. In further validation analysis, three SNPs (rs7745040, rs9275295, and rs9473132) in two genes (HLA-DRB1 and CD2AP) were confirmed to be associated with KBD.

HLA-DRB1 belongs to the HLA class II beta chain paralogs. The class II molecule is a heterodimer comprising an alpha (DRA) and a beta chain (DRB), both anchored in the cell membrane. This molecule is central to the immune system because it presents peptides derived from extracellular proteins. OA and RA have been associated with individual immune response. HLA-DRB1 has been associated with OA [16], RA [14]–[16], and KBD [17]. The finding of this study correlates with that of previous studies, which supports the association of HLA-DRB1 with KBD as well as the autoimmune disease hypothesis of KBD [17]. Seven deleterious calling SNPs were found in the HLA-DRB1 exome area based on the results of whole-exome sequencing (Table S3). Further studies on HLA-DRB1 in KBD and on the functional characterization of these SNPs are needed.

CD2AP is a 639-amino acid adapter protein, which is a scaffolding molecule expressed in all tissues except in the brain that regulates the actin cytoskeleton [18]. The protein harbors three SH3 domains in its N terminus, a proline rich domain and a C-terminal leucine-zipper domain via which it can homodimerize [19]. Common variations of CD2AP have been associated with kidney problems and late-onset Alzheimer’s diseases [20], [21], in which CD2AP was found to be involved in the abnormal cytoskeletal structural regulation and cell–cell contacts [22], [23]. In KBD, studies on proteomics and transcriptional expression profiles show far-ranging abnormal of cytoskeletal structural [4], [24]. KEGG pathway analysis indicates a large range of abnormal genes distributed in the pathway of regulation of the actin cytoskeleton (Table S2). The CD2AP gene is also involved in the lymphocyte signaling, which is connected with T cell immune response. However, whether CD2AP variations are associated with the change in the cytoskeletal structural of KBD patients as well as the function of CD2AP in KBD require further investigation.

In the first stage of this study, the whole-exome sequencing of 19 individuals showed a number of important signals in other chromosomal regions or genes, except for those discussed above. However, the follow-up investigation in independent replication did not confirm the significant association. First, the most significant p-value sites concentrated on the 19q13.2 (chr19: 38742846-54254388), which contain the SELV and TRNAU1 gene. The biological functions of SELV have been less characterized, but a redox function has been suggested [25], [26]. TRNAU1 may be associated with KBD, given that a mouse model demonstrated that the deletion of SelenocysteinetRNATrsp may result in pathological features of KBD [27]. Epidemiological investigation of environmental risks has shown that selenium deficiency may contribute to the etiopathogenesis of KBD [28]. This association was not identified in the current study possibly because of the small sample size in the validation study or the false-positive results in the first stage. Second, previous studies have shown that inflammatory factors, such as IL-1β, IL-6, and TNF-α, are involved in the pathological mechanisms of KBD. Similarly, in the current study, IL4R, CD22, CD82, CD163L1, CD180, IL12A, HLA-F-AS1, CD2AP, and HLA-DPB1 genes were found to be associated with KBD. Considering the association of HLA-DRB1 with KBD, two SNPs (rs7745040 and rs9275295) in HLA-DRB1 were chosen for the second stage of this study because of the significant p-value found in HLA-DPB1 gene located near HLA-DRB1, considering the possibility of linkage disequilibrium. Results indicate that two genes (HLA-DRB1and CD2AP) were identified to be positively associated with KBD. For other unidentified inflammatory factors, genes may show a false-positive association in the first stage because genes that are related to the immune system show far-ranging variations in human beings. Third, KEGG pathway analysis indicated that numerous genes concentrated on the regulation of actin cytoskeleton, focal adhesion, and metabolic pathways. Studies on transcription and protein expression profiling implied that expression of numerous genes and related proteins can be associated with these pathways [17], [27], [28]. However, no positive association was found in the present validation study. These pathways might represent far-reaching human physiological and metabolic pathways without any KBD-specific performance.

In this study, the susceptibility genes of KBD with whole-exome sequencing technology were comprehensively investigated. The findings obtained in this study can provide first-hand genetic information for the identification of the etiological mechanism of complex diseases. However, a number of limitations should be considered in relation to present results. First, the statistical power of this study is relatively low due to the small sample size either in the stage of whole-exome sequencing or in the replication stage in case-control analysis. Only 19 samples were used for the whole-exome sequencing, which is relatively small. The small size of the sample may provide false-positive results in the TDT test in the first stage of this study and may also increase the difficulty in selecting SNPs for the validation study. Second, only 44 SNPs distributed in 41 genes were chosen and tested in the validation stage. Although the selection of these genes was based on previous studies, which indicated that such genes/loci were found to cause KBD, the possible susceptibility of other genes cannot be excluded. Future studies should increase the sample size in whole-exome sequencing, and further studysuch as functional analysis of these genes should be carried out in order to identify therole of susceptibility genes in KBD.

In summary, HLA-DRB1 and CD2AP were identified to be the susceptibility genes of KBD, thus supporting the role of autoimmune response in KBD and the possibility of shared etiology between OA, RA, and KBD.

## Supporting Information

Figure S1 **181,870 of these SNP loci were used in the TDT test, resulting 108 SNPs with p-value<0.01 and 1056 SNPs with p-value<0.05.** This figure shows every SNP distribution in chromosome. The vertical axis represents log value after TDT test, and the horizontal axis represents 22 autosomes of human beings.
(TIF)

Table S1 **44 SNPs from 41 genes were employed for validation.**
(XLSX)

Table S2 **KEGG pathway analysis indicates that many genes with significant P value in the test focus on three major pathways.**
(XLSX)

Table S3 **Sequencing of HLA-DRB1 gene analysis found seven deleterious SNP distributed in exome area, this table listed all genotype distribution in 19 samples in the first stage of research.**
(XLSX)

Table S4 **All significant SNPs with P value<0.05 from the TDT test of exome sequencing.**
(XLSX)
