# Supplementary figures and images for: Whole-Exome Sequencing for the Identification of Susceptibility Genes of Kashin–Beck Disease
Source: PLoS One. 2014 Apr 28;9(4):e92298. doi: 10.1371/journal.pone.0092298 (PMC4002427; doi:10.1371/journal.pone.0092298)

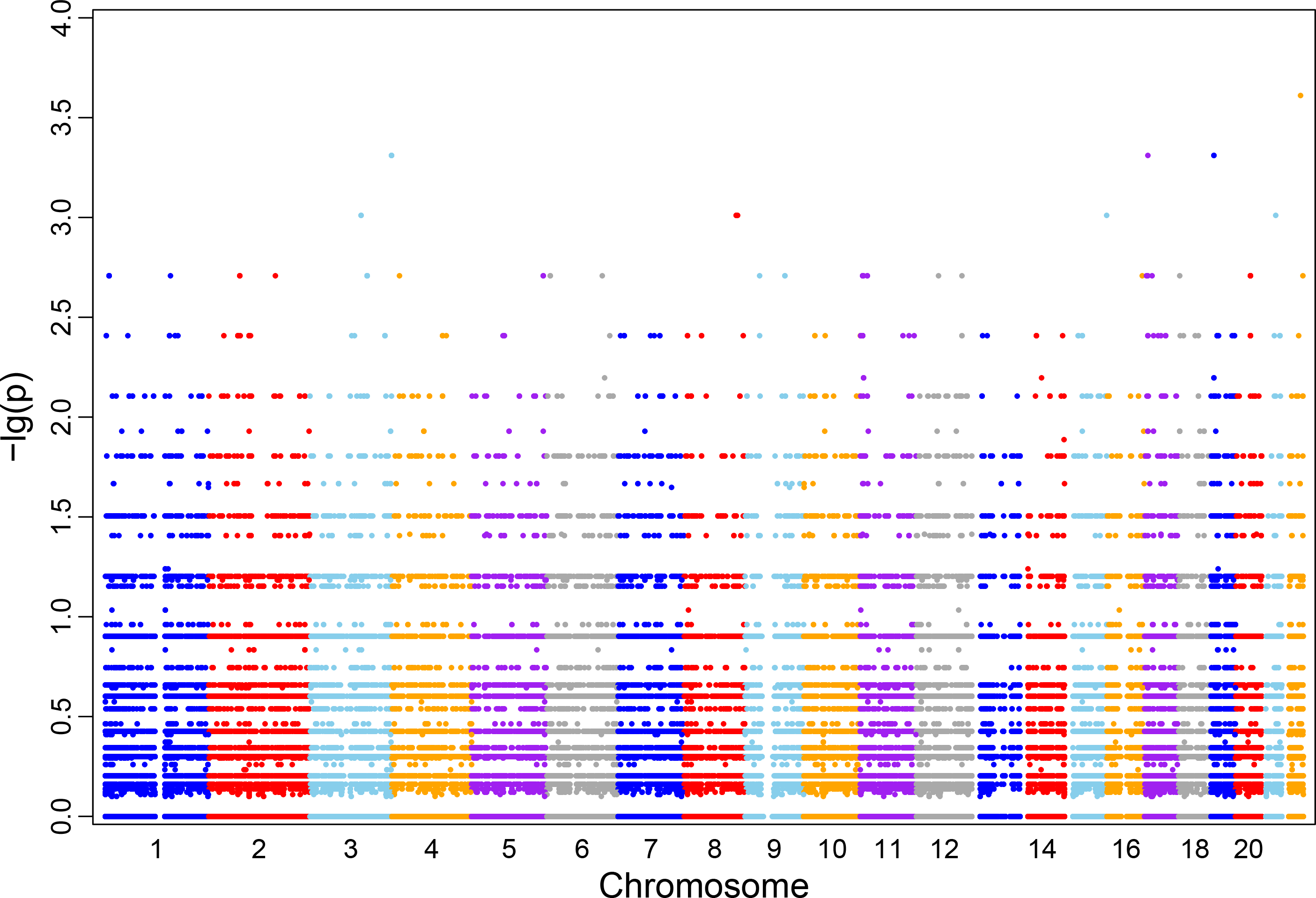

Supplement: Figure S1 — 181,870 of these SNP loci were used in the TDT test, resulting 108 SNPs with p-value<0.01 and 1056 SNPs with p-value<0.05. This figure shows every SNP distribution in chromosome. The vertical axis represents log value after TDT test, and the horizontal axis represents 22 autosomes of human beings. (TIF) [file pone.0092298.s001.tif]
